# *Fusarium* Infection in a Kidney Transplant Recipient Successfully Treated with Voriconazole

**DOI:** 10.1155/2018/3128081

**Published:** 2018-08-07

**Authors:** Ahmed M. Alkhunaizi, Ali M. Bazzi, Ali A. Rabaan, Elwaleed A. Ahmed

**Affiliations:** ^1^Nephrology Section, Specialty Medicine Department, Johns Hopkins Aramco Healthcare, Dhahran, Saudi Arabia; ^2^Department of Pathology, Johns Hopkins Aramco Healthcare, Dhahran, Saudi Arabia; ^3^Infectious Diseases Section, Specialty Medicine Department, Johns Hopkins Aramco Healthcare, Dhahran, Saudi Arabia

## Abstract

*Fusarium* infections in solid-organ transplant recipients are rare and carry high mortality. We report a case of a kidney transplant recipient who developed infection with *Fusarium* species. The patient received treatment with oral voriconazole for five months with good response.

## 1. Introduction

Opportunistic infections are common complications in patients with hematologic malignancies, bone marrow transplant patients, and recipients of solid-organ transplants [1]. Invasive fungal infections are particularly serious complications and carry a high mortality rate in immunocompromised hosts [2, 3]. *Fusarium* species are ubiquitous molds that thrive in soil and decomposing vegetation. Infection with *Fusarium* species among solid-organ transplant recipients is an uncommon event. In this report, we describe the case of a kidney transplant recipient who developed infection with *Fusarium* species and was successfully managed with voriconazole.

## 2. Case Report

**Figure 1:**
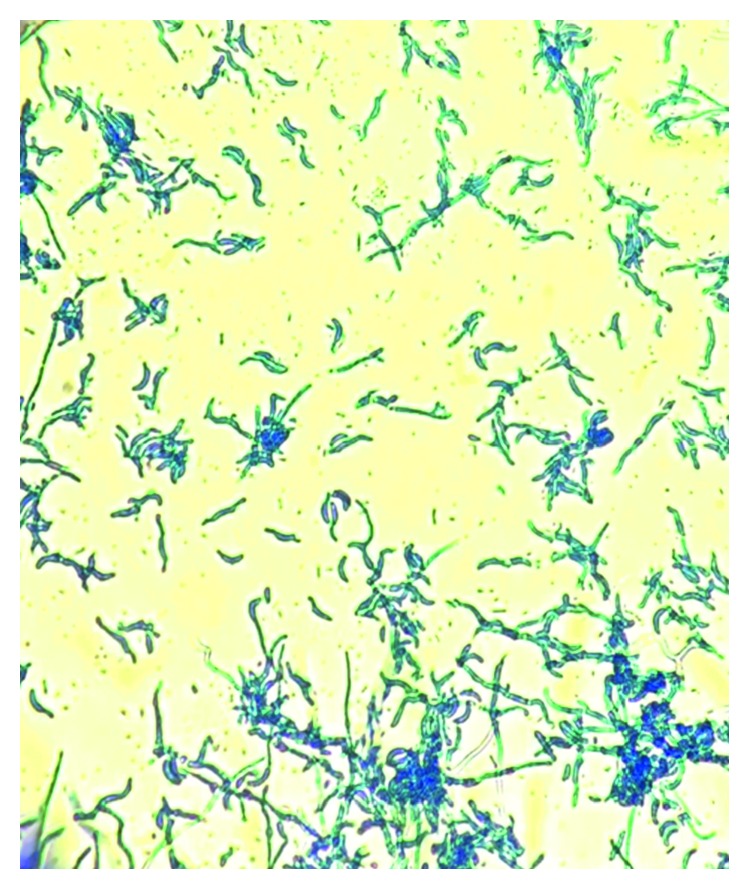
Sickle-shaped, multicell macroconidia (40x) of *Fusarium* species stained with lactophenol cotton blue counterstain from a perinephric fluid collection.

A 55-year-old male patient presented with a history of hypertension and type 2 diabetes mellitus. He developed end-stage renal disease (ESRD) and was started on hemodialysis in July 2016. In August 2016, he underwent a living unrelated kidney transplantation in another country. He received induction therapy with antithymocyte globulin and was subsequently maintained on a triple immunosuppressive regimen consisting of tacrolimus, mycophenolate mofetil, and prednisone. His postoperative course was complicated by the development of a perinephric hematoma, acute tubular necrosis, and urinary tract infection with extended-spectrum beta-lactamase producing *Escherichia coli*. Evacuation of the hematoma was performed using percutaneous drainage. In January 2017, he developed fever and abdominal pain localized to the kidney graft. Ultrasonography showed complex fluid collection surrounding the kidney measuring 20 × 9 × 8 cm. A percutaneous drain was inserted and revealed purulent fluid. Gram stain of the fluid showed many white cells and rare Gram-positive bacilli. Calcofluor white stain and KOH method showed fungal elements. The fluid culture was positive for *Fusarium* species (Figure 1).

There were no skin lesions, and the blood culture was negative. The immunosuppression regimen was modified with discontinuation of mycophenolate mofetil and reduction of the dose of tacrolimus. In addition, the patient was started on voriconazole at a maintenance dose of 4 mg/kg every 12 hours. After completing a treatment period of five months, the infection was considered cured and voriconazole was discontinued. Repeat ultrasound showed remnant cavity with septations. No adverse events were noted during the treatment period. The patient remained free of recurrent infection, and the kidney graft function remained stable with a serum creatinine of 0.9 mg/dl (normal range 0.7–1.2) 18 months after transplantation.

## 3. Discussion

Our patient developed infection with *Fusarium* species. The infection was localized to a perinephric cavity as a result of perinephric hematoma. The presence of a percutaneous drain in the setting of heavy immunosuppression in the early post-renal transplantation period was a predisposing factor. There was no other tissue or organ involvement, and therefore, we could not establish tissue invasion histologically.

Reduction of the dose of immunosuppressive medications together with a prolonged administration of voriconazole led to a cure and preservation of the renal allograft function. Susceptibility testing was not performed; however, the response to treatment indicates that the organism was sensitive to voriconazole.

Fungal infections, localized or invasive, are serious complications following organ transplantation. The consequences include prolonged hospitalization, allograft dysfunction, and high mortality, in addition to the high cost of treatment [4, 5]. Most of the fungal infections are caused by *Candida* species followed by aspergillosis and cryptococcosis [5]. The overall incidence during the first year after transplantation has been estimated at 3%; however, this varies depending on the type of organ transplantation [5]. Risk factors are related to the net state of immunosuppression, environmental exposure/colonization, and use of prophylactic antifungal agents. We have previously reported series of fungal infections related to transplant tourism similar to this case [4]. Transplant tourism is unfortunately still practiced in some parts of the world despite the commitment of many countries to the Declaration of Istanbul that aimed to combat this type of practice [6].

*Fusarium* species are ubiquitous, widely distributed in air, water, soil, and subterranean and aerial plant parts, and may occasionally infect animals and humans [7, 8]. In humans, they can cause a broad spectrum of infections including skin, eye, locally invasive infections, and in immunocompromised hosts, disseminated infections which carry a high mortality rate [2, 9]. Portals of entry include the respiratory and the gastrointestinal tracts, catheter tips, indwelling central venous catheters, and cutaneous sites [8, 10]. The most frequent species causing infections in humans are *Fusarium solani*, *Fusarium oxysporum*, and *Fusarium moniliforme* [8]. Most of the affected individuals are either bone marrow transplant recipients or have bone marrow suppression as a result of hematologic malignancy [11–14]. In contrast, infection with *Fusarium* species has been rarely reported in kidney transplant recipients, and most of the cases were related to skin infection (Table 1) [15–19]. Skin lesions can result either from direct skin invasion or through hematogenous spread in case of disseminated disease. The diagnosis of *Fusarium* infection may be made on histopathology, Gram stain, tissue culture, blood culture, or serology. Obtaining a susceptibility profile is not always possible, due to the nature of fusarial species and the inoculate size and prolonged incubation period during the susceptibility testing [20, 21]. However, all attempts should be made to perform susceptibility testing either locally or by sending the specimen to a center with experience in fungal diseases.

Treatment of fusariosis depends on the site and extent of infection. While surgical intervention may play an important role in cases of localized diseases, invasive fusariosis is more difficult to treat and poses a challenge to clinicians. Amphotericin B is effective in vitro against *Fusarium* species and historically was used to eradicate disseminated fusarial infection [22]. However, its use is limited by side effects such as nephrotoxicity especially in renal transplant recipients. More recently, voriconazole, the first available second-generation triazole, has added a new and improved therapeutic option as a primary therapy of invasive fungal infections such as aspergillosis, yeast infections, and other molds [23]. It has been used alone or in combination with other antifungal agents such as amphotericin B and terbinafine with success in treating fusarial infection [3, 24–30]. The good bioavailability of voriconazole and favorable safety profile make it easy to administer orally for a prolonged period of time. As an azole antifungal agent, voriconazole is known to inhibit cytochrome P450 3A enzymes. Drug-drug interaction with calcineurin inhibitors (CNIs) such as tacrolimus or cyclosporine is a very important consideration in treating organ transplant recipients. For example, voriconazole has been shown to inhibit the metabolism of tacrolimus by 50% [31]. Therefore, close monitoring of the blood concentration and adjustment of the dose of CNI are warranted when transplant patients receiving CNI are treated with voriconazole.

## Figures and Tables

**Table 1:** *Fusarium* infection in kidney transplant recipients.

Reference	Age (years)	Gender	Site of infection	Time at diagnosis	Treatment	Outcome
Young and Meyers [15]	30	F	Localized, skin	5 years after transplantation	Surgical excision	Cure
Heinz et al. [16]	45	M	Localized, skin	21 weeks after transplantation	Surgical amputation	Cure
Girardi et al. [17]	50	M	Localized, skin	2 years after transplantation	Surgical debridement	Poor response
Cocuroccia et al. [18]	53	M	Localized, skin	4 years after transplantation	Itraconazole	Cure
Garbino et al. [19]	56	F	Peritoneal cavity	Pretransplantation	Voriconazole for 2 months	Cure
Present report	55	M	Perinephric	One month	Voriconazole for 5 months	Cure

## References

[1] Fishman J. A. (2007). Infection in solid-organ transplant recipients. *New England Journal of Medicine*.

[2] Nucci M., Anaissie E. J., Queiroz-Telles F. (2003). Outcome predictors of 84 patients with hematologic malignancies and *Fusarium* infection. *Cancer*.

[3] Muhammed M., Anagnostou T., Desalermos A. (2013). *Fusarium* infection: report of 26 cases and review of 97 cases from the literature. *Medicine*.

[4] Alkhunaizi A. M., Amir A. A., Al-Tawfiq J. A. (2005). Invasive fungal infections in living unrelated renal transplantation. *Transplantation Proceedings*.

[5] Pappas P. G., Alexander B. D., Andes D. R. (2010). Invasive fungal infections among organ transplant recipients: results of the Transplant-Associated Infection Surveillance Network (TRANSNET). *Clinical Infectious Diseases*.

[6] International Summit on Transplant Tourism and Organ Trafficking Convened (2008). The Declaration of Istanbul on organ trafficking and transplant tourism. *Clinical Journal of the American Society of Nephrology*.

[7] Nucci M., Anaissie E. (2007). *Fusarium* infections in immunocompromised patients. *Clinical Microbiology Reviews*.

[8] Nelson P. E., Dignani M. C., Anaissie E. J. (1994). Taxonomy, biology, and clinical aspects of *Fusarium* species. *Clinical Microbiology Reviews*.

[9] Nucci M., Anaissie E. (2002). Cutaneous infection by *Fusarium* species in healthy and immunocompromised hosts: implications for diagnosis and management. *Clinical Infectious Diseases*.

[10] Gupta A. K., Baran R., Summerbell R. C. (2000). *Fusarium* infections of the skin. *Current Opinion in Infectious Diseases*.

[11] Boutati E. I., Anaissie E. J. (1997). *Fusarium*, a significant emerging pathogen in patients with hematologic malignancy: ten years’ experience at a cancer center and implications for management. *Blood*.

[12] Hennequin C., Lavarde V., Poirot J. L. (1997). Invasive *Fusarium* infections: a retrospective survey of 31 cases. *Medical Mycology*.

[13] Gamis A. S., Gudnason T., Giebink G. S., Ramsay N. K. (1991). Disseminated infection with *Fusarium* in recipients of bone marrow transplants. *Reviews of Infectious Diseases*.

[14] Campo M., Lewis R. E., Kontoyiannis D. P. (2010). Invasive fusariosis in patients with hematologic malignancies at a cancer center: 1998–2009. *Journal of Infection*.

[15] Young C. N., Meyers A. M. (1979). Opportunistic fungal infection by *Fusarium oxysporum* in a renal transplant patient. *Medical Mycology*.

[16] Heinz T., Perfect J., Schell W., Ritter E., Ruff G., Serafin D. (1996). Soft-tissue fungal infections: surgical management of 12 immunocompromised patients. *Plastic and Reconstructive Surgery*.

[17] Girardi M., Glusac E. J., Imaeda S. (1999). Subcutaneous *Fusarium* foot abscess in a renal transplant patient. *Cutis*.

[18] Cocuroccia B., Gaido J., Gubinelli E., Annessi G., Girolomoni G. (2003). Localized cutaneous hyalohyphomycosis caused by a *Fusarium* species infection in a renal transplant patient. *Journal of Clinical Microbiology*.

[19] Garbino J., Uckay I., Rohner P., Lew D., Van Delden C. (2005). *Fusarium* peritonitis concomitant to kidney transplantation successfully managed with voriconazole: case report and review of the literature. *Transplant International*.

[20] Azor M., Gene J., Cano J., Guarro J. (2007). Universal in vitro antifungal resistance of genetic clades of the *Fusarium solani* species complex. *Antimicrobial Agents and Chemotherapy*.

[21] Paphitou N. I., Ostrosky-Zeichner L., Paetznick V. L., Rodriguez J. R., Chen E., Rex J. H. (2002). In Vitro activities of investigational triazoles against *Fusarium* species: effects of inoculum size and incubation time on broth microdilution susceptibility test results. *Antimicrobial Agents and Chemotherapy*.

[22] Wolff M. A., Ramphal R. (1995). Use of amphotericin B lipid complex for treatment of disseminated cutaneous *Fusarium* infection in a neutropenic patient. *Clinical Infectious Diseases*.

[23] Boucher H. W., Groll A. H., Chiou C. C., Walsh T. J. (2004). Newer systemic antifungal agents : pharmacokinetics, safety and efficacy. *Drugs*.

[24] Consigny S., Dhedin N., Datry A., Choquet S., Leblond V., Chosidow O. (2003). Successful voriconazole treatment of disseminated *Fusarium* infection in an immunocompromised patient. *Clinical Infectious Diseases*.

[25] Ho D. Y., Lee J. D., Rosso F., Montoya J. G. (2007). Treating disseminated fusariosis: amphotericin B, voriconazole or both?. *Mycoses*.

[26] Stanzani M., Vianelli N., Bandini G. (2006). Successful treatment of disseminated fusariosis after allogeneic hematopoietic stem cell transplantation with the combination of voriconazole and liposomal amphotericin B. *Journal of Infection*.

[27] Liu J. Y., Chen W. T., Ko B. S. (2011). Combination antifungal therapy for disseminated fusariosis in immunocompromised patients: a case report and literature review. *Medical Mycology*.

[28] Inano S., Kimura M., Iida J., Arima N. (2013). Combination therapy of voriconazole and terbinafine for disseminated fusariosis: case report and literature review. *Journal of Infection and Chemotherapy*.

[29] Stempel J. M., Hammond S. P., Sutton D. A., Weiser L. M., Marty F. M. (2015). Invasive fusariosis in the voriconazole era: single-center 13-year experience. *Open Forum Infectious Diseases*.

[30] Carneiro H. A., Coleman J. J., Restrepo A., Mylonakis E. (2011). *Fusarium* infection in lung transplant patients: report of 6 cases and review of the literature. *Medicine*.

[31] Zhang S., Pillai V. C., Mada S. R., Strom S., Venkataramanan R. (2012). Effect of voriconazole and other azole antifungal agents on CYP3A activity and metabolism of tacrolimus in human liver microsomes. *Xenobiotica*.

